# Towards Control of *Streptococcus iniae*

**DOI:** 10.3201/eid1512.090232

**Published:** 2009-12

**Authors:** Justice C.F. Baiano, Andrew C. Barnes

**Affiliations:** The University of Queensland, Brisbane, Queensland, Australia

**Keywords:** Streptococcus iniae, aquaculture, pathogen, Streptococcus pyogenes, streptococci, GAS, virulence, infection, bacteria, synopsis

## Abstract

*Streptococcus iniae* is an emerging zoonotic pathogen; such infections generally occur through injuries associated with preparing whole fresh fish for cooking. Those infected to date have been of Asian descent, are usually elderly (average age 68 years), and have had >1 underlying conditions that may predispose them to infection. Studies of the foundations of growth characteristics of *S. iniae* and its interactions with piscine host cells have recently been complemented by molecular studies. Advances in molecular biology have allowed research groups to identify numerous virulence factors and to explore their roles in the progression of *S. iniae* infection. Many of these virulence factors are homologous to those found in the major human pathogen *S. pyogenes.* An increased understanding of the properties of these factors and their effect on the success of infection is leading to novel approaches to control *S. iniae* infection; in particular, vaccination programs at fish farms have reduced the reservoir of infection for additional clinical cases.

## Abstract 2

Recently identified virulence factors can lead to new approaches, such as vaccination programs at fish farms.

*Streptococcus iniae* is a major fish pathogen in many regions of the world. These bacteria are also zoonotic with infections in humans associated with the handling and preparation of infected fish. The first human infections were reported in 1996 (*1*), and *S. iniae* was noted as an emerging zoonotic disease transmitted by food animals at the International Conference on Emerging Infectious Diseases in 2000 (*2*). Human infections with *S. iniae* have been sporadic but continue to be reported with new cases arising in 2009 (*3*). Reports of these cases are likely to increase because of enhanced awareness, more reliable detection and identification methods, and the global expansion of finfish aquaculture. Most cases of human *S. iniae* infections have been in persons of Asian descent, who are elderly and commonly have >1 underlying conditions such as diabetes mellitus, chronic rheumatic heart disease, cirrhosis, or other conditions (*1*,*3*–*7*).

Carrier fish have been implicated in fish-to-fish transmission of *S. iniae* (*8*), and these carriers may be responsible for human infection because fish with overt signs of disease are unmarketable. Soft tissue injuries that occur during the preparation of fresh fish from wet markets usually result in bacteremic cellulitis of the hand, followed by >1 of these conditions: endocarditis, meningitis, arthritis, sepsis, pneumonia, osteomyelitis, and toxic shock (*7*). Infections are treated with a course of antimicrobial drugs such as penicillin, ampicillin, amoxicillin, cloxacillin, cefazolin, and/or gentamicin, doxycycline, and trimethoprim/sulfamethoxazole over a period of 1 to several weeks, depending on the nature of the infection (*3**–**5*,*9*). *S. iniae* is not currently assigned to any Lancefield group and is β-hemolytic on blood agar, with some clinical strains isolated from Asia being more mucoid than others (*6*).

Underreporting of human cases is likely because identification of *S. iniae* is based on biochemical testing of isolates with commercial kits; the use of kits is associated with problems because *S. iniae* is not listed in commercial or clinical databases, and many atypical strains are assigned low matches (*1*,*4*). According to the Australian Institute of Health and Welfare (www.aihw.gov.au), between 1999–2000 and 2006–2007, a total of 2,824 cases of “other” or “unspecified” streptococcal sepsis required hospitalization in Australia that were attributed to nongroup A, B, or C streptococci, or *S. pneumoniae,* and 2,026 cases were in persons >50 years of age. During the same period, the trend in the number of cases per year attributable to other or unspecified streptococci has been upward, rising from a total of 278 cases in 1999–2000 to 430 in 2006–2007 (155% increase). In the >50 years age group, this upward trend is more pronounced, with a 168% increase in cases requiring hospitalization. It is therefore probable that some cases of *S. iniae* infection in Australia in the at-risk age group have been misidentified. Misidentification of *S. iniae* infection is likely to be the main reason for low levels of detection because most cases of this emerging pathogen are detected during retrospective studies specifically targeting *S. iniae*. This finding is likely to be the case in countries around the world that have reported outbreaks in fish farms, but no human cases to date. Molecular-based detection and identification methods have recently been developed (information on this aspect of identification can be found in a recent review by Agnew and Barnes) and these will lead to improved reporting in future years (*10*).

Observations on the epidemiology and pathogenesis of *S. iniae* infections are still ongoing; however, valuable information on the differentiation of strains (as being either commensal or pathogenic) has benefitted research. Because of the lack of potential virulence factors or phenotypic differences between commensal and pathogenic strains, pulsed-field gel electrophoresis (PFGE) analysis showed that differences existed between human clinical isolates and those from fish surfaces (*1*). The human clinical isolates showed little variation between one another, while considerable differences were found between the 2 American Type Culture Collection (Manassas, VA, USA) dolphin strains and 32 other fish isolates. It was determined that some unknown factors important to pathogenicity were not present in all strains (*1*). Little variation has also been found between clinical strains from the United States and Canada, although 1 strain from Pennsylvania had a PFGE pattern similar to the type strain (*11*), and 2 clinical isolates from Hong Kong were unrelated to a strain from Canada (*4*). In a PFGE study of isolates from Australia, similarities between fish pathogenic isolates and human clinical isolates from North America, in addition to multiple genotypes between and within different fish farms, were reported (*12*). Another PFGE study of strains from a variety of species of diseased fish in the People’s Republic of China found that there were 17 genotypes from 27 strains clustering into 5 major groups (*13*). As with the study from Australia, multiple genotypes were found between and within different farms.

Clinical isolates from the United States were able to multiply by 2 to 5 generations in 3 hours in fresh human blood; however, 2 isolates from Canada were able to survive, but not multiply, in human blood (*11*). Resistance to phagocytic killing in whole blood by pathogenic strains of *S. iniae* contrasted with isolates identified as commensal strains that were susceptible (*9*). The pathogenic strain 9117 (a human clinical isolate causing cellulitis) caused weight loss in experimentally infected mice and was highly cellulytic to human brain microvessel endothelial cell (BMEC) monolayers and invasive of Hep-2 cells. However, adherence to, and invasion of, BMEC cells by strain 9117 was lower than that for commensal strain 9066 (obtained in a swab sample from a healthy fish) (*9*). In a similar study, pathogenic strains were more resistant to oxidative burst activity in macrophages (*14*).

The mode of invasion of *S. iniae* was studied in skin epithelial cell monolayers of rainbow trout viewed under polarized light (*15*). *S. iniae* adhered to, then invaded epithelial cells, but its persistence and replication inside the cells was short-lived. However, transcytosis from epithelial cells occurred within 30 minutes of contact without damaging the cells or cellular junctions (*15*). Once the epithelial cell layer had been breached, dissemination throughout the fish through internalization in macrophages can occur. By inducing macrophage death, this process is one of the most efficient ways of transporting the infection into the brain (*15*,*16*).

Recent molecular research into factors that contribute to the virulence of *S. iniae* has identified several candidates, including surface proteins, capsular polysaccharides, and extracellular secreted products (Figure). Moreover, the recent sequencing of the complete genome of *S. iniae* will accelerate the discovery of additional virulence factors and lead to identification of targets for effective vaccines for farmed fish, thus reducing the potential for zoonotic infection. In light of the recent rapid increase in our knowledge of this emerging pathogen, we will present a synopsis of the processes involved in infection that have been elucidated to date.

**Figure:**
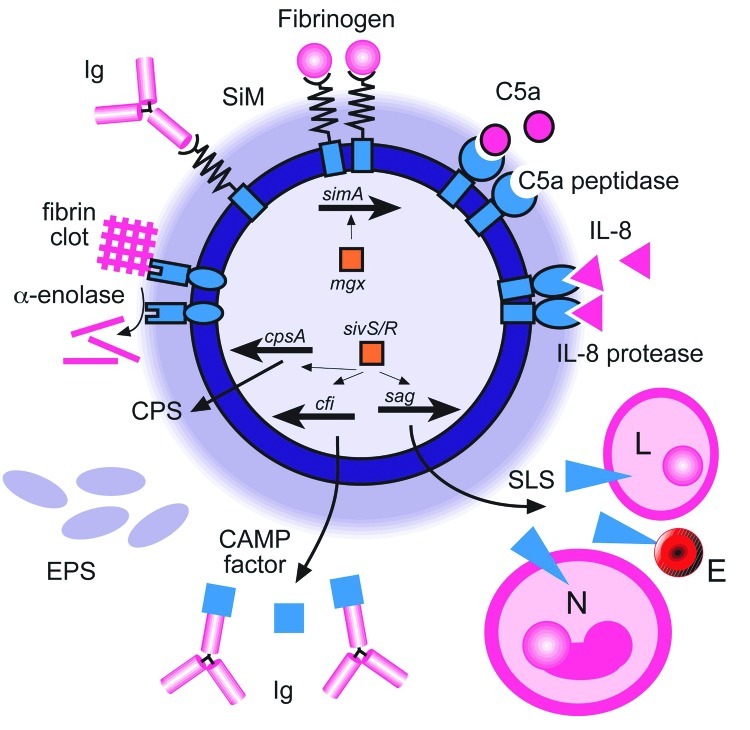
Virulence factors of *Streptococcus iniae*. A diagrammatic representation of a cell of *S. iniae* showing the regulatory genes involved in virulence factor expression (inside cell) and the virulence factors on the outside of the cell. In a clockwise direction, SiM protein (*simA*) expression is likely to be regulated by *mgx.* SiM protein binds immunoglobulin (Ig) and fibrinogen. C5a peptidase and interleukin-8 (IL-8) protease degrade their respective chemokines to impair phagocyte signaling. Production of the cytolysin streptolysin S (*sag*; SLS) is regulated by the *sivS/R* system. SLS lyses lymphocytes (L), erythrocytes (E), and neutrophils (N). The CAMP factor gene, *cfi,* is also regulated by *sivS/R* and is known to bind immunoglobulin by the Fc region. Capsular polysaccharide (*cpsA*; CPS) synthesis is controlled by *sivS/R* and is represented by a haze around the cell. Exopolysaccharide (EPS) is produced in excess and contributes to highly viscous growth. α–enolase degrades fibrin clots and promotes dissemination.

## Virulence Factors

### SiM Protein

M proteins are one of the major virulence factors in group A streptococci (*S. pyogenes*; GAS). The high level of diversity of *emm* gene types (a hypervariable gene encoding the M protein) has contributed to the success of GAS in causing infections in humans. The M-like protein from *S. iniae*, SiM, is also a prime candidate in virulence (*17*,*18*). The SiM protein is a coiled-coil protein that has a molecular mass of ≈53 kDa, although 2 other variants, one with a 1-aa insertion between the coils and another with a much larger mass of ≈59 kDa, have also been described (*17*). An additional variant had a natural frameshift/premature termination of the SiMA1 type in a strain from an infected tilapia (*18*). All SiM proteins possess the classical gram-positive membrane anchor motif LPXTG, and although they have several repeat motifs in the coil regions, they are not in tandem as in M proteins from other species. In common with M proteins from *S. pyogenes*, *S. equi,* and *S. dysgalactiae*, SiM protein is a surface protein that binds human fibrinogen to protect the bacterium from phagocytic activity (*17*). SiM proteins may also bind trout immunoglobulin by the Fc region (*19*). An allelic exchange study showed that SiM protein is a major virulence factor of *S. iniae*, contributes to adherence to fish epithelial cells (*18*), and also confirmed earlier observations that SiM contributes to macrophage resistance (*17*).

The SiM protein genes, *simA* and *simB*, are likely to be regulated by a multigene regulatory protein, Mgx, that is homologous to the Mga protein found in GAS (*17*). There are sequence elements upstream of the *simA* and *simB* genes that are similar to DNA binding sites described for Mga. The finding of a second *mga*-like element (*mgx2*) immediately downstream of *mgx* in a tilapia brain abscess isolate may represent part of an alternative virulence strategy (*18*).

### C5a Peptidase

C5a peptidase hydrolyses the neutrophil chemoattractant complement factor C5a (*18*) and thus impairs the ability of the infected host to fight the infection. C5a peptidase is a surface protein with a LPXTN gram-positive anchor motif (*18*). In GAS, C5a peptidase is found in culture supernatants; however, this observation has not been made for *S. iniae* (*18*). In *S. iniae*, C5a peptidase is a 123-kDa protein, encoded by *scpI*, with similar structural features and conserved residue positions to the GAS counterpart (*18*). C5a peptidase may have arisen in *S. iniae* by horizontal gene transfer, given its close proximity to a transposase and similar genetic organization found in GAS (*18*). Notably, allelic exchange has shown that this protein by itself is not required for virulence in fish and its role in pathogenesis is likely a minor one (*18*).

### Interleukin-8 Protease

Interleukin-8 ( IL-8) is produced in the host in response to stimuli such as lipopolysaccharides, viruses, and other cytokines. IL-8 protease is a cell envelope protease that is able to degrade the chemokine IL-8 and results in increased neutrophil resistance and disease dissemination (*20*). It is encoded by the *cepI* gene, resulting in a 1,631-aa protein with a C-terminal LPXTG gram-positive anchor motif and is homologous to the *cepA* gene in GAS (*20*).

### Streptolysin S

The ability of *S. iniae* to hemolyse erythrocytes and damage host cell membranes results from the activity of cytolysins (*21*). The cytolysin possessed by *S. iniae* is homologous to streptolysin S (SLS) from GAS (*21*) and affects erythrocytes, neutrophils, lymphocytes, and some tissue types in tissue culture (*22*), but does not have roles in phagocytic resistance nor epithelial cell adherence and invasion (*23*). Nine genes in the *sag* operon are involved in SLS formation; these share 73% homology with GAS SLS genes (*21*). The number of genes and their genetic order are identical in both microorganisms (*21*). The *sagA* gene encodes a peptide that is 73% identical to the sagA protein from GAS, and the *sagB* gene encodes a protein with 77% identity to the sagB protein from GAS, which is predicted to use flavin mononucleotide as a cofactor (*21*). The *sagC-F* genes are similar to their counterpart genes in GAS, and the *sagG-I* genes encode ATP binding cassette-type (ABC) transport systems (*21*). Other sequence features, such as inverted repeats between the *sagA* and *sagB* genes and after the *sagI* gene, have similarities with GAS *sag* operon genes (*21*). All genes in the operon are required to produce SLS as knockout of *sagB* in *S. iniae* caused loss of hemolytic activity (*21*). Likewise, when the *S. iniae sagA* gene was transformed into a nonhemolytic allelic mutant strain of GAS (NZ131 *sagA∆cat*), the *S. iniae* version of the *sagA* gene restored hemolytic activity (*21*,*23*). The cytotoxic properties of SLS toward fish cells and the likely promotion of cerebrovascular trauma represent a major virulence factor in the pathogenesis of *S. iniae* (*23*).

The *sagA* gene in *S. iniae* is regulated by a 2-component signal transduction system called *sivS/R* (*24*). *sivS/R* regulates virulence in vivo because no deaths occurred in a mouse infection model when mice were infected with a deletion mutant of pathogenic strain 9117 (9117∆*siv*) compared with 75% deaths when mice were infected with the wild type strain (*24*). In the *siv* deletion mutant, *sagA* expression was decreased by 3-fold. *sivS/R* also regulates the expression of surface proteins, including a lipoprotein/ABC transporter homologous to Spy1228, pyruvate kinase, and a hyaluronate-associated protein homologous to that from *S. equi* or an ABC transporter from *S. agalactiae* (*24*). However, the role for these proteins in a virulence setting is hitherto unknown in *S. iniae*.

### CAMP Factor

The pore-forming toxin CAMP factor synergistically acts with sphingomylinase-producing *Staphylococcus aureus* to produce a distinct arrow-shaped area of complete lysis of erythrocytes on sheep blood agar (*24*). CAMP factor has also been shown to bind immunoglobulin by the Fc region and therefore contributes to virulence (*24*). *S. iniae* harbors a CAMP factor–like gene, *cfi*, (*24*) that encodes a peptide of ≈27 kDa and shares 62% identity with *cfa* from GAS that is regulated by the *sivS/R* system. Knockout mutants of the *sivS/R* system resulted in a reduced lytic reaction, and real-time PCR analysis of *cfi* gene expression showed it was expressed at only 10% of wild-type levels (*24*).

### Immunoglobulin-Binding Proteins

A putative protein G-like protein, a cell wall associated protein first identified from group G streptococci (≈70 kDa), from *S. iniae* was capable of binding trout immunoglobulin only when grown in the presence of trout serum (*19*). Proteins of ≈35 kDa, ≈70 kDa, and >100 kDa were found to bind trout immunoglobulin. The size of one of the detected bands >100 kDa is similar in size to the tetramer formation of SiM proteins (*17*); however, experimental evidence is needed to confirm this. CAMP factor is also known to bind immunoglobulin (*24*).

### Capsule

One of the most effective ways for a bacterium to avoid phagocytosis is by the production of capsular polysaccharide (CPS), and strains with CPS are more virulent than their unencapsulated counterparts (*25*–*27*). The presence of capsule is also involved in inhibiting complement C3 deposition (*27*). Miller and Neely (*27*) used signature tagged mutagenesis to identify virulence genes using a zebrafish (*Danio rerio*) model. Five attenuated mutants with unique insertions in polysaccharide synthesis genes with homology to those found in *S. thermophilus* plus 2 additional clones with insertions in a homologous gene near capsule synthesis genes from *Bacteroides thetaiotaomicron* were found. In contrast to the wild-type strain 9117, these mutants aggregated in broth culture with chain lengths up to 4× longer than the wild type. Differences in buoyancy due to degree of encapsulation showed that the production of excess capsule is likely to be as detrimental to survival as too little capsule (*27*).

The capsule operon of *S. iniae* is ≈21 kb in size and consists of 21 open reading frames (*28*). The genes have homology to genes found in other streptococci such as *S. pyogenes, S. agalactiae, S. suis,* and *S. thermophilus* (*28*). An insertion sequence, IS*981*, was found between *cpsL* and *cpsM* in strain 9117 (*28*). *cpsY* (78% identity to CpsY in GAS) precedes *cpsA* and is transcribed in the opposite direction and is thought to be the promoter of the capsule operon (as well as other virulence genes) because it has a high level of homology to the LysR transcriptional regulators (*28*). A major difference was found in the operons from the virulent strain 9117 and commensal strain 9066 with strain 9066 having an ≈10-kb deletion missing the genes *cpsF-L* and *orf276, orf193*, and *orf151*. In addition, the *cpsM* gene in the commensal strain 9066 was truncated with the first 154 nt being absent, which casts doubt on the functionality of the gene (*28*).

The genes *cpsA-E* are responsible for the length of the monosaccharide sugar chains and their export (*29*,*30*). The central region of the operon from *cpsF* to *cpsL* contains several genes encoding glycosyl transferases, which have a role in the polymerization of the capsule chain (*28*). This region is where most mutations in the virulent strain 9117 were found to play a role in pathogenesis (*27*) and is the same region where the deleted genes in the commensal strain 9066 occurred (*28*). The G + C content of the operon genes varies widely from 22% to 40% (*28*). The *cpsF-L* region has a G + C content of ≈27%, which is lower than that found for other *S. iniae* sequences and may indicate horizontal acquisition from other members of the *Firmicutes* (*28*).

Mutations in *cpsA* in strain 9117 resulted in long chain formation, aggregation of cells in broth culture and high buoyancy characteristic of reduced encapsulation (*28*). The *cpsA* mutant was attenuated in both brain and heart tissues. A *cpsY* mutant was slightly less encapsulated than the wild type and was attenuated only in heart tissue (*28*).

Allelic exchange of the *cpsD* gene resulted in reduced capsule, increased chain length, a marked decrease in all capsular monosaccharides, and a high degree of attenuation (*31*). *cpsD* encodes an autophosphorylating tyrosine kinase thought to be responsible for capsule polymerization and export (*31*). *cpsD* mutants were able to bind more effectively to host tissues, such as epithelial cells, due to loss of overall negative charge (*31*). Insertions in the *cpsH* and *cpsM* genes have resulted in underproduction of capsule and overproduction of capsule, respectively (*28*). In a flounder isolate obtained in Japan, insertions in *cpsH*, *cpsM*, *cpsI*, and *orf276* of strain NUF631 resulted in attenuation, which was measured by increased chemiluminescence response of macrophages and loss of acidic polysaccharides (*26*). Like CAMP factor and streptolysin S, the 2-component system *sivS/R* is involved in transcriptional regulation of the capsular operon (*32*).

### Phosphoglucomutase

Also involved in capsular biosynthesis is phosphoglucomutase, a 571-aa protein encoded by *pgmA* that interconverts glucose 6-phosphate and glucose 1-phosphate (*33*). Transposon mutagenesis of the promoter region upstream of *pgmA* resulted in a highly attenuated mutant that was more susceptible to whole blood killing (*33*). This susceptibility was attributed to a decrease in the amount of exopolysaccharide capsule on the cell surface, decreased negative charge, and a larger cell volume 3–5× that of the wild type. Increased susceptibility to the pore-forming cationic antimicrobial peptide moronecidin was also reported, most likely due to changes in cell wall architecture because of increased cellular volume and a decrease in cell wall rigidity (*33*).

### Exopolysaccharide

The quantitative composition of monosaccharides present in exopolysaccharide (EPS) is distinct from those found in CPS (*34*). The routine vaccination of fish in Israel has given rise to new strains of *S. iniae* responsible for mass fish deaths (*34*). These new strains were formed when an autogenous vaccine strain, KFP404, was succeeded by new strains KFP468, KFP477, and KFP523, which were characterized by a viscous broth culture similar to that observed with *S. thermophilus* used in yoghurt production (*34*). EPS production by the successor strains was 5× higher than the autogenous vaccine strain. Vaccination of fish with the EPS extracts elicited a survival rate of 78%, which was similar to the 72% survival rate when whole cells were used. Thus, EPS appears to be antigenic and excessive production may have been selected by vaccination (*34*).

### α-Enolase

The ability of *S. iniae* to cross tissues through plasminogen activation is facilitated by α-enolase (*35*), which is also a known contributory factor to the virulence of GAS (*36*). The proteolytic activity of plasmin in dissolving fibrin clots enables pathogens to migrate faster through extracellular matrices (*37*), and α-enolase expedites invasion through the host tissues (*38*) and, ultimately, into the circulatory system. The α-enolase from *S. iniae* (≈50 kDa) is a plasmin/plasminogen binding enzyme that is 97% similar to the α-enolases of *S. agalactiae* and GAS (*35*). Immunoblot using antibodies raised against the purified recombinant protein showed cell wall association in *S. iniae*; however, it does not contain the classical gram-positive membrane anchor.

## Conclusions

*S. iniae* opportunistically infects elderly persons with serious underlying conditions. The expression of a suite of virulence factors, many of them similar to those found in GAS, is responsible for successful entry, propagation, and evasion of immune defenses of the host by this bacterium. Another virulence factor, polysaccharide deacetylase, encoded by the *pdi* gene, has been recently described (*39*). With the global rise of aquaculture and the dependence on it to provide food in many areas of the world, the numbers of cases of *S. iniae* infection are likely to be much higher than currently reported and will increase in the future with the expansion of the industry. Understanding the pathogenic processes of *S. iniae* is already facilitating the development of vaccines for use in fish farms and represents the most sustainable and effective method of reducing the incidence of economically devastating outbreaks and clinical presentations in humans, especially in those most at risk.
